# Structural distinctions in BMPs underlie divergent signaling in spinal neurons

**DOI:** 10.1186/1749-8104-7-16

**Published:** 2012-05-04

**Authors:** Jeanette C Perron, Jane Dodd

**Affiliations:** 1 Department of Physiology and Cellular Biophysics, Columbia University, 630 West 168th Street (BB1103), New York, NY, 10032, USA; 2 Department of Neuroscience, Columbia University, 630 West 168th Street (BB1103), New York, NY, 10032, USA

## Abstract

**Background:**

In dorsal spinal neurons and monocytes, bone morphogenetic protein (BMP)7 activates distinct transduction pathways, one leading to inductive specification and the other to axon orientation and chemotaxis. BMP7-evoked induction, also stimulated by the closely related BMP6, acts through a Smad cascade, leading to nuclear signaling, and is not BMPR subunit selective. Orientation is evoked by BMP7, but not by BMP6, through PI3K-dependent cytoskeletal activation mediated by the type II BMPRs, ActRIIA and BMPRII and is independent of the Smad cascade. The responses can be stimulated concurrently and suggest that BMP7, but not BMP6, can selectively activate BMPR subunits that engage the divergent paths. Although structural and biochemical analyses of selected BMP/BMPR interfaces have identified key regions of interaction, how these translate into function by related BMPs is poorly understood. To determine the mechanisms underlying the distinct activities of BMP7 and the disparate properties of BMP7 and BMP6 in spinal cord development, we have performed a family-wide structure/function analysis of BMPs and used the information to predict and test sites within BMPs that may control agonist properties, in particular the ability of a BMP to orient axons, through interactions with BMPRs.

**Results:**

We demonstrate that whereas all BMPs can induce dorsal neurons, there is selectivity in the ability also to orient axons or evoke growth cone collapse. The degree to which a BMP orients is not predictable by overall protein similarity with other BMPs but comparison of sequences of potent and weakly orienting BMPs with that of the non-orienting BMP6 revealed three candidate positions within the BMPs at which the amino acid residues may confer or obstruct orienting ability. Residue swapping analysis has identified one residue, *Gln*^*48*^ in BMP6, that blocks axon orienting ability. Replacing *Gln*^*48*^ with any of the amino acids present at the equivalent residue position in the orienting subset of BMPs confers orienting activity on BMP6. Conversely, swapping *Gln*^*48*^ into BMP7 reduces orienting ability. The inductive capacity of the BMPs was unchanged by these residue swaps.

**Conclusions:**

The results suggest that the presence of the *Gln*^*48*^ residue in BMP6 is structurally inhibitory for BMP/BMPR interactions that result in the activation of intracellular signaling, leading to axon orientation. Moreover, since residue 48 in BMP7 and the corresponding residue in BMP2 are important for type II BMPR binding, our results provide a basis for a mechanistic understanding of the diverse activities of BMPs in spinal cord development.

## Background

Bone morphogenetic proteins (BMPs) represent a class of TFGβ factors with diverse functions in mammals. BMPs evoke transcriptional events leading to cellular differentiation and survival [1,2] but also direct axon guidance and cellular orienting activities through cytoskeletal signaling [3-5]. The large number of BMPs, their differing affinities for an array of heteromeric BMP receptors (BMPRs), and a range of extracellular and intracellular modulators of BMP/BMPR interactions [6,7] suggest that differential expression of these BMP signaling components could produce the many actions of BMPs. However, in neurons and monocytes, a single BMP can simultaneously evoke both transcriptional and cytoskeletal responses that involve different receptor subunits and divergent intracellular signaling programs [3,8,9], suggesting additional mechanisms that supersede the simple distribution of transduction components. Moreover, the ability to activate divergent pathways is not necessarily shared by closely related BMPs, highlighting the importance of individual agonist properties [8,9]. We sought here to understand the properties that underlie the selective ability of a BMP to exert divergent orienting activity.

In the mammalian central nervous system (CNS), BMP6, BMP7 and the more distantly related GDF7, are expressed with overlapping distribution in the roof plate, at the dorsal midline of the developing spinal cord [4,10]. Roof plate BMP activity mediates the induction of dorsal spinal interneurons (dI neurons) [10,11] and the subsequent guidance of the axons of nascent dI neurons but examination of the individual BMPs uncovered different roles for these family members during spinal cord development [4,12]. Comparison of the inductive and orienting activities of BMP6, BMP7 and GDF7 in spinal explants revealed that while all three induce ectopic dI neurons, only BMP7 can also orient extending dI axons [4,10,12]. Moreover, whereas BMP6 stimulates induction but has no orienting activity in dissociated dI neurons or monocytes, BMP7 activates inductive signaling and also evokes growth cone collapse in dI neurons and chemotaxis in monocytes [4,9,12]. Nonetheless, the different activities of the closely related BMP6 and BMP7 in dorsal spinal development have remained a puzzle.

The results summarized above, combined with the finding that orienting responses to BMP7 are initiated at much lower concentrations than BMP-evoked inductive signaling in the same cells [3,8,9], led to the idea that, whereas both BMP7 and BMP6 engage receptor complexes that activate intracellular inductive machinery, BMP7 alone recruits a distinct receptor complex that directs signaling towards the cytoskeleton. In support of this, we and others have provided evidence that the type II BMPR subunits, ActRIIA and BMPRII, and the type I BMPR subunit, BMPRIB, are required selectively by BMP7 to activate cellular orienting responses of neurons and monocytes but are not individually essential for BMP inductive activity [8,13]. In recent work, both BMP7 and BMP6 have been shown to activate Smad signaling and dI neuron induction through the activity of type I BMPR kinases, whereas BMP7-evoked axonal orientation is independent of type I BMPR kinase activity [9]. Furthermore, BMP7, but not BMP6, activates a PI3K-dependent pathway required for axon orientation but PI3K-dependent signaling is not required for BMP7-stimulated induction [9]. Together, these findings suggest a model whereby the ability of BMP7 to stimulate divergent intracellular pathways within a cell results from the differential recruitment and/or activation of BMPRs, but a mechanistic explanation for such selective subunit recruitment is still lacking.

BMPs bind as dimers to BMPR complexes comprising one pair each of type I BMPR and type II BMPR subunits [6]. Classically, phosphorylation of the type I receptors by the type II receptor pair leads to stimulation of the intracellular cascade of activated Smads [1,14] resulting in transcriptional signaling. In addition, however, type II BMPRs appear to interact directly with signaling intermediates that regulate cytoskeletal events [15,16]. Variation in receptor composition and the mode of receptor subunit recruitment by different BMPs are thought to contribute to choice of distinct intracellular pathways [17,18] and this notion, combined with differences in binding potencies of BMPs to BMPRs [19-21], suggests that individual BMPs may achieve distinct cellular outcomes through differential usage of subunits in BMPR complexes.

An unresolved issue, however, is the basis for selective agonist interactions with these BMPR subunits. Structural modeling and binding studies of BMP interactions with the extracellular domains (ECD) of BMPRs have identified sites of interaction between select BMPs and type I and type II BMPRs [22-24]. Indeed, the predicted BMP7/ActRIIA and BMP2/BMPRIA interfaces are known and found in spatially distinct regions [23,25,26]. It seems likely that understanding how these regions of the BMPs contribute to their agonist properties will elucidate the mechanisms underlying selective BMP function in developing spinal cord. We have, therefore, performed a structure/function analysis across the BMP family, examining inductive, growth cone collapsing and axon orienting ability on dI neurons. We have used this information to identify features of the primary structure of BMPs that may control orienting ability and have begun to test these inferences using a residue swapping approach. Our analysis reveals one residue within the putative type II BMPR binding domain of BMP7 and BMP6 which is critical for orienting ability, while not impinging on inductive capacity. These results suggest a model for the mechanism underlying the ability of BMPs to recruit or activate selectively certain BMPR subunits leading to axon orientation signaling.

## Results

### Characterization of orienting activity across the BMP family

**Figure 1:**
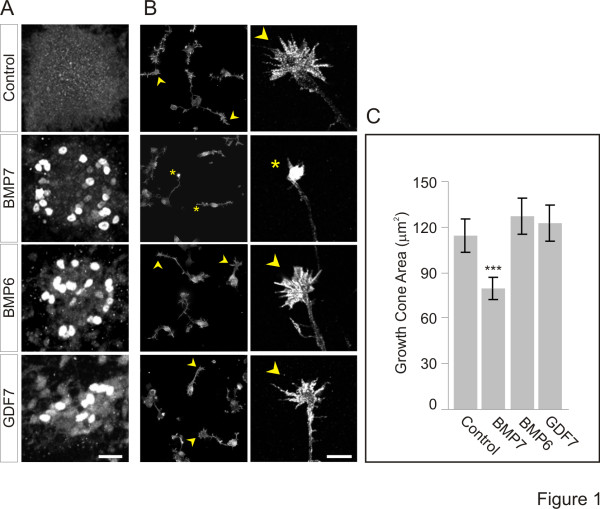
**Differential activity of roof plate-resident BMPs in induction and dI neuron growth cone collapse. (A)** Lhx2/9 induction in [i] explants exposed to 50 ng/ml rBMP7, rBMP6 and rGDF7. Scale = 25 μm. **(B)** ERM-labeled dissociated dI neurons ± rBMP7, rBMP6 or rGDF7 at 50 ng/ml. Arrowheads indicate typical widespread growth cones and asterisks indicate collapsed growth cones. Low power fluorescence images are shown in the left column (scale = 40 μm) and high power confocal images are shown in the right column (scale = 10 μm). **(C)** Average growth cone areas (mean ± SEM): Control = 114.3 ± 10.7 μm^2^; BMP7 = 79.5 ± 7.4 μm^2^; BMP6 = 127.4 ± 11.9 μm^2^; GDF7 = 122.4 ± 11.7 μm^2^. Student’s *t* tests: growth cone area measured in BMP7 is significantly different from control, BMP6 and GDF7 (***P <0.001); growth cone area in response to BMP6 or GDF7 did not differ from control (*P* = 0.0607 and *P* = 0.2105, respectively). Results are for 100 to 115 growth cones/condition/experiment; n = 2.

In addition to orienting dI axons, BMP7 has a direct and rapid effect on dissociated dI neurons, evoking growth cone collapse within minutes and providing a convenient and direct bioassay for orientation ability [4,9]. To illustrate the disparate actions of the roof plate-resident BMPs, we tested recombinant (r) BMP7, rBMP6 and rGDF7, at the same concentrations, in parallel growth cone collapse and induction assays. We used 50 ng/ml rBMPs, a concentration well above threshold for both the inducing activity of BMP6 or BMP7 (approximately 5 ng/ml) and the growth cone collapse activity of BMP7 (0.01 ng/ml; [9]). Explants of intermediate neural tube ([i] explants) were incubated in BMP then probed for the differentiation of dI1 neurons, marked by expression of the LIM homeodomain proteins, Lhx2 and Lhx9 [11,27] as previously described [9]. All three BMPs induced Lhx2/9 expression (Figure 1A), indicating that they were functional at this concentration. However, only rBMP7 reduced the area of growth cones (30% reduction; Figure 1B, C) whereas rBMP6 and rGDF7 had no effect on growth cone size (Figure 1B, C). The results are consistent with findings in which BMP7, but not BMP6 or GDF7, orients dI axons in explants of embryonic dorsal spinal cord ([d] explants) [4,9,12] and support the notion that the inability to orient axons by BMP6 and GDF7 reflects different agonist properties rather than concentration issues.

**Figure 2:**
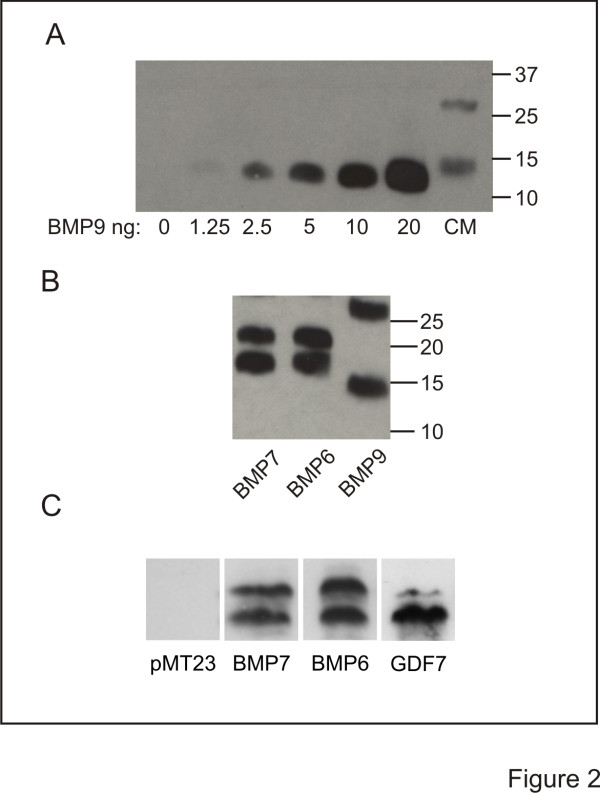
**Generation of BMP conditioned medium (CM). (A-C)** Western blots of rBMPs and 20-fold concentrated CM. **(A)** rBMP9 at the indicated concentrations and BMP9 CM probed with α-BMP9. **(B)** myc-tagged BMP CM probed with α-myc. **(C)** HA-tagged BMP CM probed with α-HA. In CM lanes, the lower bands represent BMP monomers migrating at 18 kDa for BMP7 and BMP6, 15 kDa for GDF7 and 13 kDa for BMP9. The upper bands represent glycosylated forms of the BMPs.

The disparate activity profiles of the roof plate BMPs prompted us to perform a comprehensive analysis of the dI axon orienting and dI inductive abilities across the BMP family. The activities of BMPs and several other members of the transforming growth factor (TGF)β superfamily were tested in [d] explants. In this preparation, COS-1 cell aggregates expressing epitope-tagged proteins are appended asymmetrically to the explants. The effects of expressed BMPs on orientation of TAG-1^+^ axons of endogenous dI neurons and induction of ectopic Lhx2/9^+^ dI1 neurons in [d] explants are examined concurrently [4,9,12]. We first assessed the production of BMPs and TGFβs by measuring the secretion of epitope-tagged proteins into medium conditioned by transfected COS-1 cells. Each conditioned medium (CM) was examined by Western blot, using BMP-specific antibodies or antibodies that recognize the epitope tag (HA, myc or flag). Concentrations of BMPs were estimated by comparison with Western blot standard curves for given rBMPs, shown here for BMP9 (Figure 2A). CM collected from COS-1 cells expressing myc- (Figure 2B) or HA-tagged (Figure 2C) BMPs contained similar levels of secreted protein independent of the epitope tag used, in the range of 250 to 500 pg/ml. Moreover, in this study the concentrations of all BMPs and TGFβs in CM were similar (not shown).

**Figure 3:**
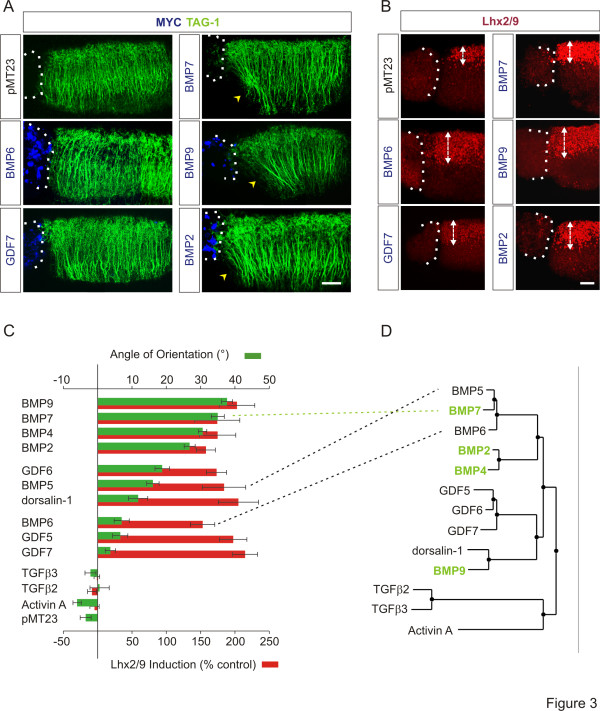
**A subset of BMPs, not predictable by structure, has orienting ability.(A, B)** [d] explants with asymmetrically appended COS-1 cell aggregates expressing myc-tagged BMPs co-labeled with α-TAG-1 (green), α-Lhx2/9 (red) and α-myc (blue). Dashed lines mark appended borders of explants and aggregates. **(A)** dI axon responses in [d] explants. Arrowheads indicate axons repelled by BMPs. Scale = 50 μm. **(B)** BMP-stimulated induction of ectopic Lhx2/9 expression in [d] explants. Double-headed arrows mark width of Lhx2/9-expression regions. Scale = 50 μm. **(C)** Histograms comparing orienting and inductive activities of BMP/TGFβs in [d] explants, grouped according to orientation activity. **Green bars** indicate angle of axonal orientation (mean ± SEM): BMP9 = 37.7 ± 1.6° (n = 31); BMP7 = 35.0 ± 1.9° (n = 22); BMP4 = 30.6 ± 1.2° (n = 14); BMP2 = 26.8 ± 1.6° (n = 20); GDF6 = 18.8 ± 2.1° (n = 22); BMP5 = 16.1 ± 1.8° (n = 28); dorsalin-1 = 11.8 ± 2.8° (n = 15); BMP6 = 7.0 ± 2.2° (n = 24); GDF5 = 6.6 ± 2.2° (n = 20); GDF7 = 3.7 ± 1.5° (n = 19); TGFβ3 = −2.1 ± 1.6° (n = 11); TGFβ2 = 0.56 ± 2.8° (n = 9); Activin A = −6.0 ± 1.2° (n = 21); pMT23 = −3.5 ± 1.6° (n = 14). Student’s *t* tests: Axon orientation responses to BMP5 and BMP6 were significantly different (*P* = 0.0022) with responses to BMP7 significantly different from both BMP5 and BMP6 (*P* <0.0001), revealing three distinct levels of orienting activity in the BMP5/6/7 subgroup. **Red bars** indicate Lhx2/9 induction (mean ± SEM): BMP9 = 203 ± 26% (n = 11); BMP7 = 174 ± 33% (n = 6); BMP4 = 175 ± 26% (n = 10); BMP2 = 158 ± 14% (n = 11); GDF6 = 173 ± 15% (n = 4); BMP5 = 184 ± 31% (n = 7); dorsalin-1 = 205 ± 29% (n = 7); BMP6 = 153 ± 18% (n = 7); GDF5 = 198 ± 20% (n = 12); GDF7 = 215 ± 18% (n = 14); TGFβ3 = −8 ± 6% (n = 10); TGFβ2 = −1 ± 4% (n = 9); Activin A = −5 ± 7% (n = 16). Inductive activity is not statistically different among the BMPs (*P* = 0.5773, ANOVA). **(D)** Phylogenetic tree of BMPs and select other members of the TGFβ superfamily. BMPs with robust orienting activity are highlighted in green. Dashed lines between (C) and (D) highlight differing orienting activities yet close structural relationship of the BMP5/6/7 subgroup.

We next appended pellets of BMP- and TGFβ-expressing COS-1 cells to [d] explants and measured the orientation of TAG-1^+^ dI axons growing within the explants. In [d] explants cultured adjacent to control COS-1 cells expressing empty vector, pMT23, dI axons extended with a dorsal to ventral (D-V) trajectory (angle of orientation (pMT23): -3.5 ± 1.6°; Figure 3A and 3C (green bars)). In [d] explants with appended BMP7-expressing COS-1 cells, TAG-1^+^ axons were repelled, extending away from the BMP7 source, with an angle of orientation of 35 ± 1.9° (Figure 3A, C). Three other BMPs, BMP9, BMP4 and BMP2, also showed robust orienting activity with a range of orientation angles of 27 to 38° (Figure 3A, C; BMP9: 38 ± 1.6°; BMP4: 31 ± 1.2°; BMP2: 27 ± 1.6°). GDF6, BMP5 and dorsalin-1 showed intermediate orienting activities with a range of orientation angles of 12 to 19° (Figure 3C; GFD6: 19 ± 2.1°; BMP5: 16 ± 1.8°; dorsalin-1: 12 ± 2.8°), whereas BMP6, GDF5 and GDF7 showed little or no activity which was statistically not different from control (Figure 3A, C; range of orientation angle: 4 to 7^o^; BMP6: 7 ± 2.2°; GDF5: 7 ± 2.2°; GDF7: 4 ± 1.5°). The non-BMP, TGFβ family members, TGFβ3, TGFβ2, GDF15 and Activin A, were all inactive in axon orientation (Figure 3C and not shown; Range of orientation angle: -6 to 0.6^o^; TGFβ2: 0.6 ± 2.8°; TGFβ3: -2 ± 1.6°; GDF15: -3 ± 3.9°; Activin A: -6 ± 1.2°).

The ability of the BMPs and TGFβs to induce differentiation of ectopic dI neurons was assessed by measuring ectopic Lhx2/9 expression (Figure 3B) in the same set of [d] explants used to record axon orientation. In control [d] explants, cultured adjacent to pellets of COS-1 cells expressing pMT23, endogenous expression of Lhx2/9 was restricted to dorsal regions of the explants (Figure 3B and see [9]). In [d] explants co-cultured with BMP7-expressing COS-1 cells ectopic Lhx2/9 expression was observed (Figure 3B). The D-V extent of Lhx2/9 expression was expanded in response to BMP7, reflecting a 2.7-fold increase in dI1 neurons (Figure 3C (red bars)). All 10 BMPs showed similarly robust dI1-inducing ability (Figure 3B, C) stimulating induction of Lhx2/9^+^ cells in a range of 153 to 215% over control. None of the other TGFβs tested induced ectopic Lhx2/9 expression (Figure 3C). These results indicate that although all BMPs have dI1 neuron inducing capacity, only a subset exhibits axon orienting activity.

### Discrete structural distinctions correlate with the ability of BMPs to orient axons

BMPs fall into closely related subgroups assigned according to overall mature protein similarity (see Figure 3D). Comparison of subgroup relationships with respect to orienting activity of BMPs (compare Figure 3C and D) revealed that axon orienting BMPs are not those most highly related by overall amino acid similarities. BMP7, BMP6 and BMP5 represent the most closely interrelated subset of BMPs (Figure 3D) yet display orienting activity that can be categorized as high, insignificant and intermediate, respectively (Figure 3C (green bars)). BMP9 is relatively distantly related to BMP7 yet has the most comparable orienting ability. The capacity to induce ectopic Lhx2/9 expression was similar across the family of BMPs but did not extend to other members of the TGFβ family (Figure 3C (red bars)). Thus the ability of BMPs to induce neural character appears to be a property common to all BMPs, but the ability of BMPs to orient is restricted to a subset of active BMPs that does not mirror groupings according to overall structural similarity.

**Figure 4:**
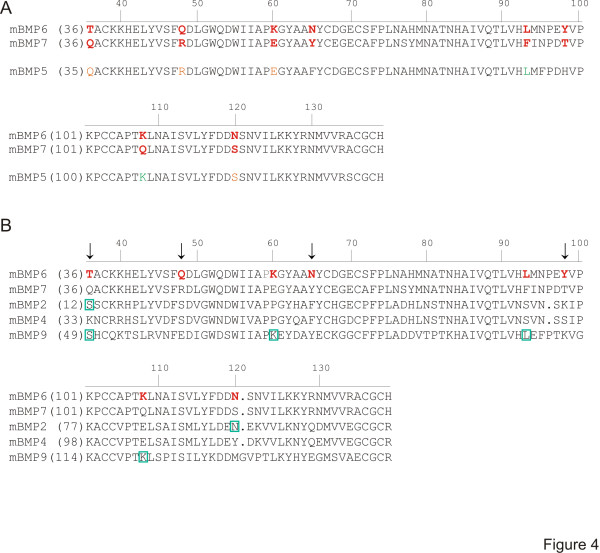
**Comparison of amino acid sequences of mature BMPs.** Murine (m) sequences are aligned beginning two amino acids upstream from the first conserved cysteine of the mature region [10] and numbered along the top according to BMP7 sequence. Numbering corresponding to individual BMPs is shown to the left of each sequence. **(A)** Comparison of BMP6 with BMP7 and BMP5. Bold red letters in BMP6 and BMP7 indicate non-conservative amino acid differences. Orange letters in BMP5 indicate residues that are the same as those in BMP7. Green letters in BMP5 represent residues the same as those found in BMP6. **(B)** Comparison of BMP6 with other BMPs that orient: BMP7, BMP2, BMP4 and BMP9. Arrows indicate amino acid residues (36, 48, 65, 98 (BMP7 numbering)) that are unique in BMP6. Each of the other four amino acid differences in BMP6 is shared by at least one other BMP with orienting activity (indicated by green boxes). At position 36, the residues in BMP2 and BMP9 (green boxes) represent a conservative substitution from that in BMP6.

To look in more detail for primary structural components that might affect the ability of BMPs selectively to activate downstream signaling leading to orientation, we aligned the amino acid sequences of the mature, secreted protein regions of BMP6 and BMP7 (Figure 4A), choosing to compare these initially because they are the most closely similar BMPs that show the maximum divergence functionally. Allowing for conservative amino acid substitutions in the sequences, BMP6 and BMP7 could be seen to have 95% similarity, with non-conservative amino acid differences at only eight positions, dispersed across the sequence (Figure 4A). Notably, BMP5, the other member of this structural subgroup and displaying intermediate orienting activity, was found to be 97% similar to BMP7 and shares with BMP7 four of the residues that are divergent in BMP6. Three of these are grouped at the N-terminal region, at positions 36, 48 and 60. But BMP5 also shares with BMP6 two of the residues, located closer to the C-terminal end of the protein, that diverge from the BMP7 sequence, at positions 93 and 108 (Figure 4A). These observations raised the possibility that residues that are shared by BMP7 and BMP5, but absent in BMP6, promote orienting activity, and/or residues selectively present in BMP6 reduce orienting activity. Nonetheless, the functional distinctions remain unresolved by this limited comparison.

To probe further the identity of residues that might confer orienting activity, we next compared the sequences of BMP6 and BMP7 with the sequences of the other BMPs with robust orienting activity. Of the eight amino acid residues that are expressed in BMP6 but not BMP7, four are also expressed in other robustly orienting BMPs (green boxes in Figure 4B) and one (residue 36) contains a conservative substitution between BMP6 and BMPs2/9. Thus these five residues seem unlikely to represent positions critical for orienting activity. The three remaining divergent residues, at positions 48, 65 and 98 in BMP6, are not present in the corresponding positions in any of the BMPs with orienting ability, including BMP5, designating these residues as candidate determinants of orienting ability. We therefore next examined the influence of residues 48, 65 and 98 in BMP6 and BMP7 to confer or depress orienting activity.

### Residue swapping selectively alters BMP orienting ability

**Figure 5:**
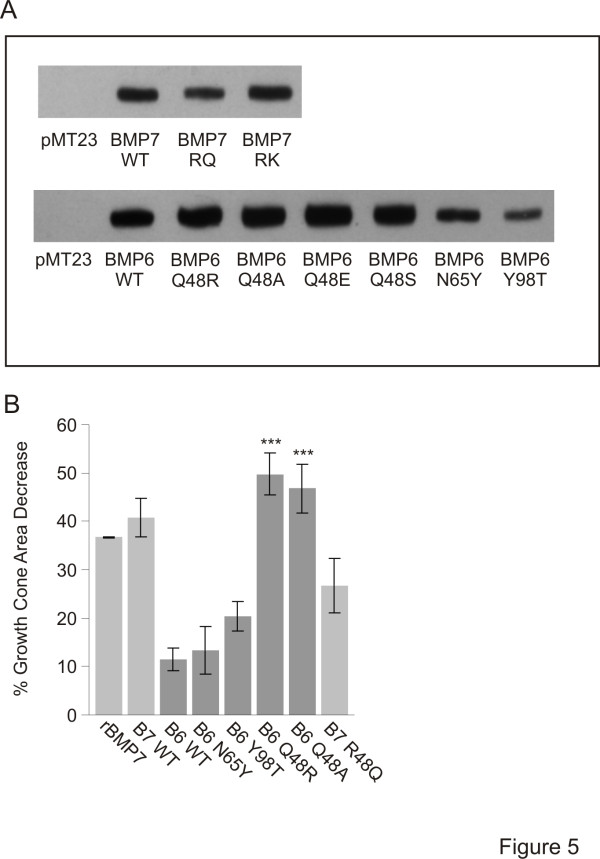
**BMP6/BMP7 chimeras exhibit dramatically different growth cone collapse activity from that of BMP6 WT. (A)** Western blot analysis of whole cell lysates of COS-1 cells expressing control vector (pMT23) and HA-tagged native (WT) and chimeric BMP constructs. The bands represent the 60 kDa BMP proprotein. The fully processed form is secreted into the COS-1 cell medium which is used as CM in growth cone collapse assays. **(B)** Growth cone collapse activity of WT and chimeric BMPs. Percentage decrease of growth cone area relative to control cultures (mean ± SEM): rBMP7 = 36.7 ± 0.3%; BMP7 WT = 40.7 ± 4.0%; BMP6 WT = 11.5 ± 2.3%; BMP6 N65Y = 13.3 ± 4.9%; BMP6 Y98T = 20.4 ± 3.1%; BMP6 Q48R = 49.8 ± 4.5%; BMP6 Q48A = 46.8 ± 5.0%; BMP7 R48Q = 26.7 ± 5.7%. Student’s *t* tests: BMP6 Q48R- and BMP6 Q48A-evoked reduction of growth cone area is significantly different from BMP6 WT treated cultures (***P = 0.0004 and ***P = 0.0007, respectively). BMP6 Q48R- and BMP6 Q48A-stimulated growth cone collapse is not different from BMP7 WT treated cultures (*P* = 0.2600 and *P* = 0.4425, respectively). Results are for 100 to 250 growth cones/condition/experiment; n = 2.

To test the functional importance of residues 48, 65 and 98 in BMP6 and BMP7, we generated single amino acid substitutions individually in the BMP6 sequence to the corresponding residues in BMP7 and examined the ability of BMP6/BMP7 chimeras to evoke growth cone collapse, axon orientation and induction. Glutamine (Q) at position 48 in BMP6 (*Gln*^*48*^) was substituted with arginine (generating BMP6 Q48R), asparagine (N) at position 65 (*Asn*^*65*^) substituted with tyrosine (BMP6 N65Y) and tyrosine at position 98 (*Tyr*^*98*^) substituted with threonine (BMP6 Y98T). Unaltered (WT) and chimeric BMPs were each HA tagged and expressed in COS-1 cells. Western blot analysis of lysates, using α-HA antibodies, demonstrated that all BMPs were expressed abundantly (Figure 5A).

We first examined the abilities of BMP6/BMP7 chimeras to evoke dI neuron growth cone collapse, comparing them with the activities of BMP6 WT, BMP7 WT and rBMP7. Dissociated dI neurons in sister cultures were incubated with either COS-1 cell CM or rBMP7 and changes in growth cone area were assessed as in Figure 1B, C. BMP7 WT CM caused reduction of growth cone area that was not significantly different from that evoked by rBMP7 (41 ± 4.0% BMP7 WT; 37 ± 0.2% rBMP7, compared to control CM from COS-1 cell cultures expressing vector alone; Figure 5B). BMP6 WT CM did not cause significant growth cone collapse (12 ± 2.3% reduction in growth cone area; Figure 5B). Similarly, BMP6 N65Y and BMP6 Y98T CM were ineffective in evoking growth cone collapse, showing no improvement over BMP6 WT CM (13 ± 4.9% and 20 ± 3.1% reduction in growth cone area, respectively; Figure 5B). In contrast, BMP6 Q48R CM caused a decrease in growth cone area (50 ± 4.4% reduction in growth cone area; Figure 5B) that was similar to that stimulated by BMP7 WT CM and rBMP7. The dramatic acquisition of activity by BMP6 Q48R but not by BMP6 N65Y or BMP6 Y98T suggested that residue 48 is important for the ability of a BMP to evoke growth cone collapse.

**Figure 6:**
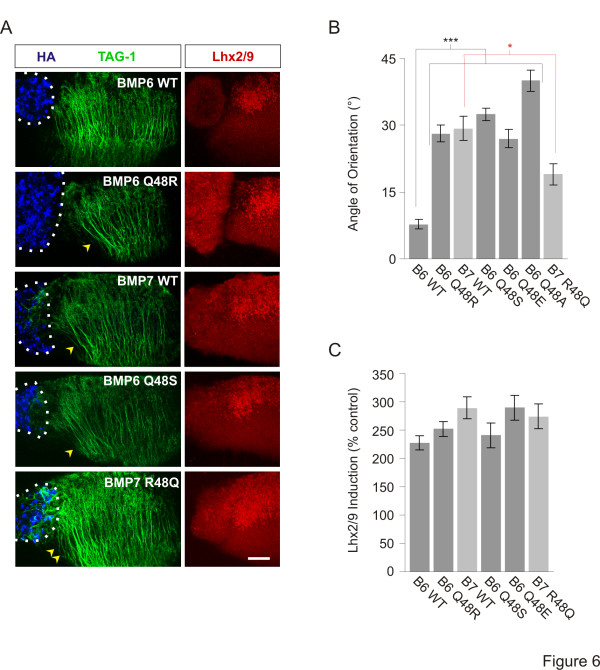
**BMP6*Gln***^***48***^**residue appears to inhibit orienting activity without affecting dI induction. (A)** Aggregates of COS-1 cells expressing HA-tagged native (WT) and chimeric BMPs appended to [d] explants co-labeled with α-HA (blue), α-TAG-1 (green) and α-Lhx2/9 (red). Dashed lines mark appended borders of aggregates and explants. Arrowheads indicate dI axons repelled by BMP6 Q48R, BMP6 Q48S and BMP7 WT. Double arrowheads indicate reduced dI axon repulsion in explants exposed to BMP7 R48Q. Scale = 50 μm. **(B)** Angle of orientation in [d] explants (mean ± SEM): BMP6 WT = 7.8 ± 1.1° (n = 11); BMP6 Q48R = 28.1 ± 1.9° (n = 22); BMP7 WT = 29.3 ± 2.7° (n = 11); BMP6 Q48S = 32.5 ± 1.4° (n = 4); BMP6 Q48E = 27.0 ± 2.1° (n = 3); BMP6 Q48A = 40.0 ± 2.4° (n = 8); BMP7 R48Q = 19.0 ± 2.9° (n = 16); pMT23 = −4.8 ± 1.1° (n = 6). Student’s *t* tests: Axon orientation evoked by each BMP6/BMP7 chimera is significantly different from that evoked by BMP6 WT (***P <0.0001). Axon orientation evoked by BMP7 R48Q is significantly different from that evoked by BMP7 WT (*P <0.01). **(C)** Quantification of Lhx2/9 induction relative to control (pMT23) exposed [d] explants (mean ± SEM): BMP6 WT = 228 ± 13% (n = 11); BMP6 Q48R = 252 ± 14% (n = 16); BMP7 WT = 289 ± 19% (n = 8); BMP6 Q48S = 241 ± 22% (n = 4); BMP6 Q48E = 290 ± 22% (n = 5); BMP7 R48Q = 274 ± 21% (n = 11). Inductive activity is not statistically different between any of the native and chimeric BMPs (*P* = 0.1280, ANOVA).

We next tested the ability of BMP6 Q48R to orient dI axons in [d] explants and the results strikingly mirrored those found in the growth cone analysis: whereas BMP6 WT did not orient dI axons (7.8 ± 1.1°; Figure 6A, B), BMP6 Q48R oriented dI axons as effectively as BMP7 WT (BMP7 WT: 29 ± 2.7**°**; BMP6 Q48R: 28 ± 1.9**°**; Figure 6A, B). In contrast, the ability of BMP6 Q48R, and all other chimeras tested, to induce Lhx2/9-expressing dI neurons in the same [d] explants was unchanged in comparison to BMP6 WT (Figure 6A, C and not shown). These results suggest that residue 48 is a selective determinant of orienting ability.

### Glutamine at position 48 depresses orienting activity

The ability of BMP6 Q48R to orient axons and evoke growth cone collapse suggests either that there is a requirement for *Arg*^*48*^ (which is large and positively charged) or that *Gln*^*48*^ (which is large and uncharged) is inhibitory to the selective BMP to BMPR interactions needed for orientation activity. Inspection of the residues found in other orienting BMPs, BMP2, BMP4 and BMP9, at the position corresponding to *Arg*^*48*^ in BMP7 suggests that the properties of *Arg*^*48*^ may not be explicitly required to control orientation activity: BMP2 and BMP4 show serine (*Ser*) (which is small and uncharged) instead of arginine and BMP9 contains glutamic acid (*Glu*) (which is of medium size and negatively charged) at this position (Figure 4B). Nonetheless, *Arg*, *Ser* and *Glu* may each confer orienting activity. To test this, we first asked whether swapping *Ser* or *Glu* into position 48 in BMP6 would confer orienting activity. Both BMP6 Q48S and BMP6 Q48E showed strong orienting activity (33 ± 1.4° and 27 ± 1.4°, respectively; Figure 6A, B and not shown), appearing as effective as BMP6 Q48R and BMP7 WT. As with BMP6 Q48R, both BMP6 Q48S and BMP6 Q48E showed similar inductive activity to that of BMP6 WT (Figure 6A, C).

To determine whether the acquisition of orienting activity by BMP6 Q48R, Q48S and Q48E chimeras reflects a requirement specifically for any one of the three amino acids found in other orienting BMPs, we replaced *Gln*^*48*^ in BMP6 with a generic amino acid, alanine (A). BMP6 Q48A evoked robust growth cone collapse (47 ± 5.0% reduction in growth cone area; Figure 5B) and oriented dI axons within [d] explants (40 ± 2.4°; Figure 6B), showing similar responses to those evoked by BMP6 Q48R. Together, these results suggest that the beneficial effect of residue swapping into position 48 may result from removal of *Gln*^*48*^ rather than a selective positive effect of *Arg*, *Ser* or *Glu*. The inability of BMP6 to orient might be determined by *Gln*^*48*^ preventing interaction between BMP6 and a BMPR at a critical interface. To test the possibility that the *Gln*^*48*^ residue negatively regulates activity in BMP6 we therefore generated a reciprocal BMP7 construct in which *Arg*^*48*^ in BMP7 was replaced with *Gln* (BMP7 R48Q). Strikingly, BMP7 R48Q had substantially reduced growth cone collapse activity (27 ± 5.7% reduction in growth cone area; Figure 5B) and dI axon repulsion (19 ± 2.4°; Figure 6A, B) by comparison with BMP7 WT activity, whereas inductive activity in the same explants was unchanged (Figure 6A, C). These results suggest that *Gln*^*48*^ determines the inability of BMP6 to orient axons.

## Discussion

Here we have addressed an unexplained aspect of agonist specificity in BMP signaling, a feature that may control the different functions of highly related BMPs during early spinal cord development. We have explored the mechanism by which two highly related BMPs have dramatically different abilities to activate signaling that regulates cytoskeletal dynamics, leading to axon orientation, although sharing the ability to evoke inductive signaling in the same cell. That these distinct responses can be activated by a single BMP and appear to depend on different receptor subunit activation led us to examine the BMP family for agonist properties that might influence selective BMP/BMPR interactions. We show that the activities of the whole family recapitulate the disparate functions of the roof plate BMPs. Whereas all BMPs share the ability to induce dI neuron differentiation, only a subset of the family have axon orienting activity and this subset is not predicted by overall sequence similarity. Exploiting the close similarities of the BMP family, however, we identified amino acid residues as candidates for conferring orienting ability on BMPs. Generation of chimeric BMPs by single amino acid substitutions has revealed a critical position at which the residue confers or reduces orienting activity but does not influence inductive activity. Although this critical residue is unlikely to be the sole determinant of BMP orienting activity, it lies within the predicted interface between BMP7 and ActRIIA, a receptor required for orienting activity. These results provide mechanistic insight into the ability of BMPs to recruit distinct receptor complexes and elicit different functional outcomes.

### Comparative analysis reveals residue 48 as critical for orienting activity

Identification of BMPs as “orienting” or “non-orienting” was fundamental to the structure/function analysis presented here and two lines of evidence provided confidence that all BMPs were tested well within the functional concentration range for orientation. First, in Western blots of COS-1 cell lysates and CM similar levels of efficient expression were observed for all native and chimeric constructs, indicating that residue swapping did not interfere with BMP processing or secretion. Second, in COS-1 cell CM, BMPs were expressed in 25-fold excess of the concentration required for BMP7 to evoke orientation [9]. We and others have shown that while neural induction and activation of associated downstream signaling components requires high concentrations of BMPs, the orienting activities of BMP7, including growth cone collapse and monocyte chemotaxis, occur at considerably lower concentrations [3,4,9]. The finding that all 10 native BMPs and each chimeric BMP induced the ectopic expression of Lhx2/9 in [d] explants, and to a similar extent, suggests that explants were exposed to comparable concentrations of BMPs. Thus the observed absence of axon orienting activity in BMP6 WT and BMP6 N65Y and Y98T chimeras, as well as the reduction in axon orienting activity in the BMP7 R48Q chimera, reflects an inability to activate the relevant pathways through specific agonist properties and leads to the conclusion that residue 48 in BMP6 and BMP7 influences receptor binding in a critical manner.

### Residue 48 is unlikely to be the sole determinant of orienting ability

Single amino acid substitutions in BMPs have previously been shown to cause dramatic differences in BMP activities and in BMPR binding [28-30]. The identification of the residue at position 48 as critical for the orienting activity of BMPs adds to this cannon. However, in experiments with the reciprocal chimera, BMP7 R48Q, orienting activity is reduced but not fully inhibited, indicating that residue 48 in BMP6/7 is not the only determinant of orienting activity, and that there may be subtle mitigating effects from other residues present in BMP7 but not BMP6. The existence of additional determinants is also suggested by our finding that orienting activity across the BMP family is not simply categorized in binary fashion. BMP5, most closely related in sequence to BMP7 and BMP6, and containing an arginine residue (*Arg*^*47*^) at the position corresponding to *Arg*^*48*^ in BMP7, typifies a group of BMPs with intermediate orienting activity in [d] explants. In this functional study, we chose first to examine residues in BMP6 and BMP7 that were the most different from each other (non-conservative amino acid substitutions). However, considering both conservative and non-conservative amino acid differences observed in BMP6 and BMP7 sequences, several are matched between the sequences of BMP5 and BMP6. These residues are located mainly in a region thought to be important for type I BMPR binding [23,26] and therefore may not be involved directly in specific type II BMPR interactions. Nonetheless, these and other differences present in the BMP sequences may influence the conformation of the BMP ligand and its interaction with the BMPR complex, such that graduated functional outcomes are generated. The intermediate orienting activity of BMP5 may therefore be a useful tool, in conjunction with BMP6 and BMP7, with which to identify amino acids that can confer BMP6-like or BMP7-like activity upon BMP5 and to provide a more comprehensive picture of the determinants for orienting activity mediated by BMPs.

### How does residue 48 affect BMP agonist properties?

Crystal structure studies of BMP7 bound to ActRIIA show that two of the BMP7 residues that differ in BMP6, *Arg*^*48*^ and *Tyr*^*65*^, lie within the predicted BMP7/ActRIIA interface [22,23,26]. These two residues thus represent candidate regulators of selective interactions with ActRIIA, one of the BMPR subunits that we have shown to be required for orienting activity of BMP7 [8]. Replacing residue 65 in BMP6 with *Tyr*, however, appeared not to confer orienting activity on BMP6, but coordinate multiple residue swaps might be necessary to reveal a more subtle role for residue position 65. Notably, none of the BMP6 or BMP7 chimeras altered BMP-evoked inductive signaling, revealing a singular role for residue 48 and representing a point of agonist/receptor interaction required selectively for orientation. However, our results throw into question the idea that specific amino acids are required to be present at position 48 in BMP7, BMP2/4 and BMP9 for orienting activity. Rather, *ArgSer* and *Glu* may be permissive for this activity, whereas the properties of *Gln*^*48*^ prevent BMP6 from activating the signaling apparatus that transduces orienting activity. Indeed, the potencies of BMP6 Q48R, Q48S, Q48E and Q48A in axon orientation were as high as that of BMP9, the most potent of the BMPs, whereas in BMP7 swapping of *Arg*^*48*^ to *Gln*^*48*^ reduced orienting activity dramatically.

**Figure 7:**
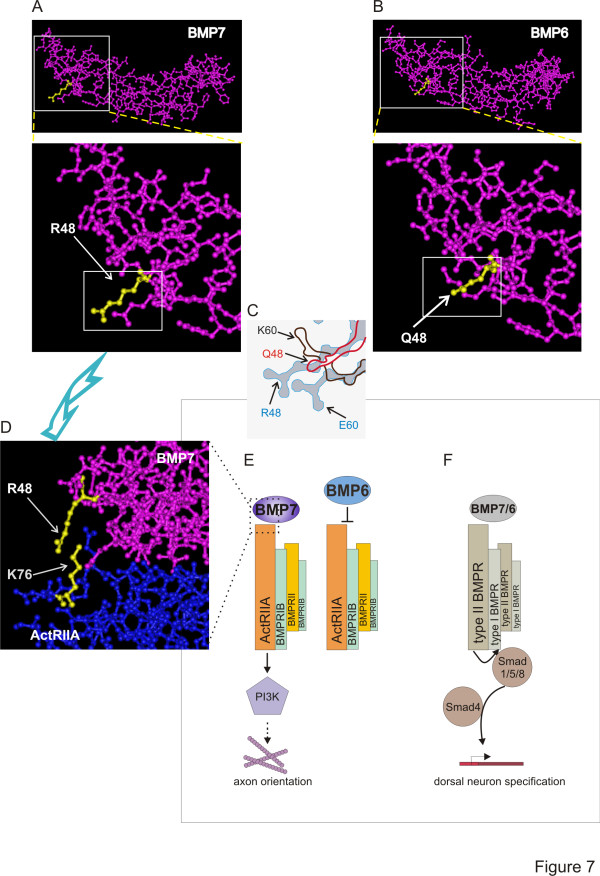
**Model of mechanism of BMP selectivity in axon orienting ability. (A-D)** Ball and stick structural representations of BMP7, BMP6 and BMP7 bound to the AcRIIA ECD. The images were generated with Cn3D v4.3 software (http://www.ncbi.nlm.nih.gov), shown at low and high magnification. **(A)** Views of the BMP7 monomer (PDB: 1LX1, [23] focusing on the region containing *Arg*^*48*^ (residue highlighted in yellow). **(B)** Views of the BMP6 monomer (PDB: 2R52, [33]) in the proximity of *Gln*^*48*^ (yellow). **(C)** Enlargement and overlay of the boxed areas in the lower panels in A and B illustrating the differences in the configuration of residues in this region: for BMP7, *Arg*^*48*^ and *Glu*^*60*^ (blue); for BMP6, *Gln*^*48*^ (red) and *Lys*^*60*^ (brown). The arrangement of residues in this region is likely to influence binding specificity and subsequent functional outcomes. **(D)** High magnification view of the BMP7/ActRIIA ECD complex (PDB: 1LX5; [23]. BMP7 is rotated with respect to the view in A to illustrate the predicted interaction of *Arg*^*48*^ in BMP7 and *Lys*^*76*^ in ActRIIA. **(E)** Axon orientation and growth cone collapse is evoked by BMP7 but not by BMP6 and involves signaling dependent on PI3K [9] and, by analogy with BMP7-evoked monocyte chemotaxis [8], likely depends on activation of ActRIIA. Our model proposes that BMP7 and other orienting BMPs are able selectively to activate a receptor complex comprising ActRIIA, BMPRII and BMPRIB (see [8,9,13]) but that *Gln*^48^ in BMP6 prevents interaction with this complex**. (F)** The induction pathway, activated by both BMP7 and BMP6, and all other BMPs, leads to stimulation of the Smad cascade and induction of target genes. This transduction mechanism has a high threshold for activity, is not selectively dependent on any of the type II BMPRs [8,9] and is unaffected by chimeric BMPs that alter orienting activity.

How might *Gln*^*48*^ reduce activity? Intriguingly, *Arg*^*48*^ in BMP7 lies within the ‘knuckle’ epitope, the major binding interface of BMPs with type II BMPRs, but is not part of the ‘wrist’ epitope, which is the main site in BMPs for type I BMPR interaction [22,31]. Indeed, both *Arg*^*48*^ in BMP7 and *Ser*^*24*^ in BMP2/4 (equivalent positions) have been implicated in high-affinity binding to type II BMPRs [22]. Furthermore, structural modeling predicts a direct interaction of *Arg*^*48*^ in BMP7 with *Lys*^*76*^ in ActRIIA (Figure 7D; [30]). However, the residue at position 60 (BMP7 numbering) has also been implicated in type II BMPR binding [23,30]. BMP7 and BMP6 differ at residue 60 (Figure 7C) but this residue was eliminated from our functional analysis because BMP9 shares *Lys*^*60*^ with BMP6. Nevertheless, the proximity of residues 48 and 60 raise the possibility that together these residues prevent BMP6 from stimulating orienting activity. Indeed, comparison of the crystal structures of BMP7 [32] and BMP6 [29,33] reveals significant 3D differences in the region of the position 48 residue (Figure 7A-C) and suggests that BMP7 has greater flexibility than BMP6 in the fingers that comprise the knuckle epitope [30]. Although BMP6 and BMP7 have been shown to have similar affinities for ActRIIA [29,33], the different properties of the amino acids at position 48 in BMP6 (*Gln*, large and uncharged) and BMP7 (*Arg*, large and charged) as well as other BMPs that can orient (*Ser*, small and uncharged; *Glu,* small and polar) (see Figure 7A-D), may affect mode of binding rather than absolute binding capacity.

Several studies have explored the importance of the mode of binding of BMPs to receptor subunits. The cellular response to BMPs has been shown to depend on the mode of receptor oligomerization [17,18,34]. BMP binding to preformed receptor complexes drives Smad-dependent, transcriptional pathways, whereas BMP-induced receptor subunit assembly leads to non-transcriptional responses, such as cytoskeletal rearrangements [17,18]. Although the presence of the type I BMPR, BMPRIB, is necessary for the orientation response [13], type I BMPR kinase activity, which is required for BMP-evoked Smad phosphorylation and inductive signaling, appears not to be important for orientation [9]. In contrast, BMP7-evoked monocyte chemotaxis requires the selective engagement of type II BMPRs, ActRIIA and BMPRII [8]. Together, these data suggest a model (Figure 7) in which BMP7 and other orienting BMPs are able selectively to recruit a receptor complex responsible for BMP orienting activity, presumably comprising ActRIIA, BMPRII and BMPRIB (Figure 7E), whereas BMP6 is unable to stimulate orienting activity through this receptor complex (Figure 7E), perhaps prevented by structural differences in which the *Gln*^*48*^ residue plays a key role in type II BMPR binding specificity. In contrast, BMP-evoked inductive activity (Figure 7F), which appears to be common to all BMPs but requires higher agonist concentrations and may reflect less stringent requirements for BMPR binding, perhaps involving preformed receptors.

## Conclusion

BMPs share the ability to induce the differentiation of dorsal spinal neurons, but only a subset exhibit orientation activity toward the axons of these neurons. The closely related BMP family members, BMP6 and BMP7 with 95% similarity, most dramatically illustrate this difference. BMP7 does and BMP6 does not display orienting activity. We show here that a single amino acid residue at position 48 in BMP7 is a major determinant of BMP orienting activity. Residue swapping of *Gln*^*48*^ in BMP6 and *Arg*^*48*^ present in BMP7 confers robust axon orienting ability upon BMP6 and reduces activity in BMP7. Moreover, replacing the *Gln*^*48*^ residue in BMP6 with the corresponding residue in other orienting BMPs permitted BMP6 to orient axons and collapse growth cones of dI neurons. In contrast, none of these manipulations altered BMP dI inductive activity. Our results suggest that the presence of the *Gln*^*48*^ residue in BMP6 is structurally inhibitory for transduction of the signals necessary for axon orientation by BMPs and provide a basis for our mechanistic understanding of the diverse activities of BMPs in spinal cord development.

## Materials and methods

### Antibodies and reagents

Recombinant BMPs were purchased from R&D Systems, Minneapolis, MN, USA, and stock solutions were prepared in 4 mM HCl/0.1% BSA. Antibodies: mouse α-TAG-1 (4D7; [35]), rabbit α-Lhx2/9 (L1; [11]), mouse α-ERM (13 H9; [36]), rabbit α-HA, mouse α-HA (Abcam, Cambridge, MA, USA), mouse α-myc (9E10; [37]), rabbit α-BMP9 (see below). HRP- and fluorophore-conjugated secondary antibodies were obtained from Jackson ImmunoResearch Laboratories, West Grove, PA, USA. Cell culture reagents: Ham’s F12 medium, Opti-MEM medium, Penicillin/Streptomycin/Glutamine (P/S/G), Penicillin/Streptomycin (P/S) (Invitrogen, Carlsbad, CA, USA), FBS (Gemini BioProducts, West Sacramento, CA, USA) and 45% glucose (Sigma-Aldrich, St Louis, MO, USA). Pharmacological reagent: LY294002 (LY; Cell Signaling Technology, Danvers, MA, USA): stock solution was prepared in DMSO and subsequently diluted in serum-free Opti-MEM/P/S/G.

The α-BMP9 polyclonal antibody was generated by immunizing rabbits with a peptide (MGVPTLKYHYEG) corresponding to C-terminal amino acids 133 through 144 in the mature coding sequence of mouse BMP9 (Covance Research Products, Inc., Denver, PA, USA). The antiserum was affinity purified using the Montage Antibody Purification Kit (Millipore Corporation, Bedford, MA, USA).

### Generation of BMP/GDF/TGFβ expression constructs

Mouse cDNA was prepared from whole E11.5 embryos with Superscript II reverse transcriptase (Invitrogen). The mature regions of all BMP/TGFβ superfamily members used in this study were generated by PCR using gene-specific primers containing either myc (EQKLISEEDL), flag (DYKDDDDK) or HA (YPYDVPDYA) epitope tag insertions and cloned into pMT23 as previously described [10,38].

### BMP mutagenesis

Single amino acid mutations were performed using the QuickChange Site-Directed Mutagenesis Kit (Stratagene, La Jolla, CA, USA). Primers were designed and used to produce nucleotide changes in the native HA-tagged mouse BMP6.pMT23 expression construct to yield the Q48R, Q48A, Q48S, Q48E, N65Y and Y98T chimeras and in the native HA-tagged mouse BMP7.pMT23 construct to yield the R48Q chimera. The sequences were verified by analysis (Genewiz, South Plainfield, NJ, USA).

### Generation and characterization of conditioned medium

COS-1 cells plated in 35 mm dishes were transfected with control vector (pMT23), epitope-tagged native or chimeric BMP expression constructs by transfection with either Lipofectamine Reagent or Lipofectamine LTX Reagent (Invitrogen). Following an overnight incubation, the medium was replaced with 2 mL serum-free Opti-MEM/P/S/G. Conditioned medium (CM) was collected after 48 hours and used in growth cone collapse assays. For analysis of secreted BMPs, CM was concentrated 20-fold with Centricon YM-3 Amicon concentrators (Millipore, Bedford, MA, USA). Concentrated CM or whole cell lysates, prepared from transfected COS-1 cells using 1x Lysis Buffer (Cell Signaling Technology,) supplemented with 1 mM PMSF, were separated by SDS-PAGE (EZ-Run Gel Solution, Fisher Scientific, Pittsburgh, PA, USA) and transferred to nitrocellulose (Whatman, Clifton, NJ, USA). Nitrocellulose membranes were blocked in 5% non-fat milk/0.1% Tween 20/TBS (Blocking Buffer) and probed overnight with epitope tag or BMP-specific antibodies diluted in Blocking Buffer. Membranes were washed in TBST (0.1% Tween 20/TBS) and probed (1 hour) with HRP-conjugated secondary antibodies in Blocking Buffer. After washing in TBST, blots were developed using the Supersignal West Pico chemiluminescent substrate detection kit (Pierce, Rockford, IL, USA) and exposed to Kodak BioMax Light Film Kodak, Rochester, NY, USA).

### Growth cone collapse assay

Dissociated dI neuron cultures were prepared as previously described [9]. The cultures were serum-starved by incubation in unsupplemented F12 medium for 2 hours at 37°C and stimulated with rBMPs at 50 ng/ml or with COS-1 cell CM (500μL) for 30 minutes. The cultures were fixed in pre-warmed 4% paraformaldehyde/0.5% gluteraldehyde/0.1 M phosphate buffer for 5 minutes, washed once in PBS, blocked in 1% heat-inactivated goat serum/0.1% Triton X-100/PBS and labeled with a mouse α-ERM IgM and a Cy3 goat α-mouse IgM secondary antibody. The growth cone area of neurons with axons greater the 10 μm was measured across two or three coverslips per condition for each experiment using ImageJ 1.37v software (National Institutes of Health (NIH)). Growth cone collapsing activity is presented as raw mean area or as the percentage decrease of growth cone area relative to control cultures.

### [i] explant assays

Intermediate spinal cord ([i]) explants were dissected from stage 10 chick embryos, cultured in collagen and immunolabeled as previously described [39,40]. BMPs (50 ng/ml) or 4 mM HCl/0.1% BSA (control) were incubated with the explants for 48 hours.

### [d] explant assays

E11 rat [d] explants were dissected, cultured and labeled as previously described [4]. COS-1 cells were transfected with epitope-tagged native or chimeric BMP expression constructs using Lipofectamine Reagent or Lipofectamine LTX Reagent (Invitrogen), aggregated [41] and appended to [d] explants as described [4]. Explants were immunolabeled with antibodies against TAG-1, Lhx2/9 and the epitope tag of the COS-1 cell-expressed BMP. Lhx2/9 induction and the angle of reorientation of TAG-1^+^ dI axons were measured in each explant in parallel. Quantitation of Lhx2/9 induction, using ImageJ (NIH), was performed by measuring the percentage change in integrated density (mean pixel intensity x area) of the BMP-induced region of Lhx2/9^+^ cells present in the explant relative to control (pMT23) explants. The angle of reorientation was measured as shown previously [4].

### Imaging

Images of dI neuron dissociated cultures were taken with a Zeiss AxioCam HR digital camera (Carl Zeiss, Thornwood, NY, USA) mounted on a Zeiss Axiovert 200 M fluorescence microscope. In addition, images of [i] and [d] explants were taken using a Zeiss LSM 5 confocal microscope and are presented here as confocal Z-stacks.

### Alignments and dendrogram

Amino acid alignments of the mature regions of BMPs were made using Vector NTI software (Invitrogen). Sequences are aligned beginning two amino acids upstream from the first conserved cysteine of the mature region [10,11]). The BMP/TGFβ phylogenetic tree dendrogram was generated using ArboDraw v1.3 software (http://dunbrack.fcccc.edu/ArboDraw). All sequences are from mouse, except for chick dorsalin-1.

## Abbreviations

ActR: Activin-like receptor; BMP: Bone morphogenetic protein; BMPR: Bone morphogenetic protein receptor; BSA: Bovine serum albumin; CM: COS-1 cell conditioned medium; [d] explants: Explant of E11 rat dorsal spinal cord; dI: Dorsal spinal interneuron; DMSO: dimethyl sulfoxide; D-V: Dorsal to ventral; E: Embryonic day where E0.5 = 6 am on the day of plug; ECD: Extracellular domain; ERM: Ezrin-radixin-moeisin FBS, fetal bovine serum; HA: Human influenza hemagglutinin; HRP: Horseradish peroxidase; [i] explant: Explant of intermediate region of spinal cord of Hamburger Hamilton Stage 10 chick embryo; PI3K: Phosphoinositide-3-kinase; P/S: Penicillin/streptomycin; P/S/G: Penicillin/streptomycin/glutamine; rBMP: Recombinant BMP; TAG-1: Transient axonal glycoprotein; TGFβ: Transforming growth factor beta; WT: Wild-type.

## Competing interests

The authors declare that they have no competing interests.

## Authors’ contributions

The work was conceived, designed and planned by JP and JD in collaboration. JP performed all experiments described here. Analysis was performed by JP and JD. The manuscript was drafted by JP and JD and both authors approve the manuscript.
